# Influence of childhood growth on asthma and lung function in adolescence

**DOI:** 10.1016/j.jaci.2014.10.046

**Published:** 2015-06

**Authors:** Agnes M.M. Sonnenschein-van der Voort, Laura D. Howe, Raquel Granell, Liesbeth Duijts, Jonathan A.C. Sterne, Kate Tilling, A. John Henderson

**Affiliations:** a School of Social and Community Medicine, University of Bristol, Bristol, United Kingdom; d Medical Research Council Integrative Epidemiology Unit, University of Bristol, Bristol, United Kingdom; b Department of Pediatrics, Division of Respiratory Medicine, Erasmus Medical Center, Rotterdam, The Netherlands; c Department of Epidemiology, Erasmus Medical Center, Rotterdam, The Netherlands; e Department of Pediatrics, Division of Neonatology, Erasmus Medical Center, Rotterdam, The Netherlands

**Keywords:** ALSPAC, asthma, cohort study, growth, lung function, ALSPAC, Avon Longitudinal Study of Parents and Children, FEF_25-75_, Forced expiratory flow between 25% and 75%, FVC, Forced vital capacity, OR, Odds ratio

## Abstract

**Background:**

Low birth weight and rapid infant growth in early infancy are associated with increased risk of childhood asthma, but little is known about the role of postinfancy growth in asthmatic children.

**Objectives:**

We sought to examine the associations of children's growth patterns with asthma, bronchial responsiveness, and lung function until adolescence.

**Methods:**

Individual growth trajectories from birth until 10 years of age were estimated by using linear spline multilevel models for 9723 children participating in a population-based prospective cohort study. Current asthma at 8, 14, and 17 years of age was based on questionnaires. Lung function and bronchial responsiveness or reversibility were measured during clinic visits at 8 and 15 years of age.

**Results:**

Rapid weight growth between 0 and 3 months of age was most consistently associated with increased risks of current asthma at the ages of 8 and 17 years, bronchial responsiveness at age 8 years, and bronchial reversibility at age 15 years. Rapid weight growth was associated with lung function values, with the strongest associations for weight gain between 3 and 7 years of age and higher forced vital capacity (FVC) and FEV_1_ values at age 15 years (0.12 [95% CI, 0.08 to 0.17] and 0.11 [95% CI, 0.07 to 0.15], *z* score per SD, respectively) and weight growth between 0 and 3 months of age and lower FEV_1_/FVC ratios at age 8 and 15 years (−0.13 [95% CI, −0.16 to −0.10] and −0.04 [95% CI, −0.07 to −0.01], *z* score per SD, respectively). Rapid length growth was associated with lower FVC and FVC_1_ values at age 15 years.

**Conclusion:**

Faster weight growth in early childhood is associated with asthma and bronchial hyperresponsiveness, and faster weight growth across childhood is associated with higher FVC and FEV_1_ values.

Asthma is the most prevalent chronic respiratory disease in children worldwide.^1,2^ Many factors have been associated with increased risk of asthma or lower lung function, such as gestational age, tobacco smoke exposure, breast-feeding habits, and a family history of asthma or allergy.^3-7^ Respiratory morbidity might also be the result of abnormal growth. Fetal growth^8-10^ and low birth weight^10-16^ have been associated with asthma. Several studies have explored the associations of infant or childhood growth with the risk of asthma or lung function in later life.^10,17-26^ They reported an increased risk of asthma symptoms in preschool children with accelerated growth in early infancy,^10,23^ an increased incidence of asthma at 6 years after a rapid increase in body mass index in early childhood,^22^ a lower FEV_0.4_ value in the first months of life in children with greater postnatal weight gain,^21^ and a negative association of growth with lung function during the first year of life.^27^ However, other studies observed no evidence for increased risk of asthma caused by rapid growth^24^ or observed that weight gain during the first year was positively associated with lung function.^19^ These inconsistencies could be explained in part by methodological issues, including differences in the definitions of growth or asthma outcomes and adjustment for potential confounders.

It was suggested in a published study that growth in early infancy, especially from birth to 3 months,^10^ might be an important influence on asthma risk. However, it is unknown whether this association persists until adolescence or influences lung function, although tracking of lung function suggests that its trajectory is established by midchildhood.^20,28^ It is also not known whether the first 3 months after birth is the only important time period or whether any specific period after the first year of age might play a role as well.

The underlying mechanism of the associations between growth and respiratory morbidity might include abnormal growth and development of the lungs or immunologic or inflammatory effects, such as adiposity-related systemic and tissue-specific inflammation.^29-33^ To test our hypothesis that rapid early growth is negatively associated with respiratory health, we examined the association of children's growth trajectories from birth until age 10 years with current asthma, bronchial responsiveness or reversibility, and lung function in adolescence in a population-based prospective birth cohort study among 9723 children.

## Methods

### Design and setting

**Fig E1:**
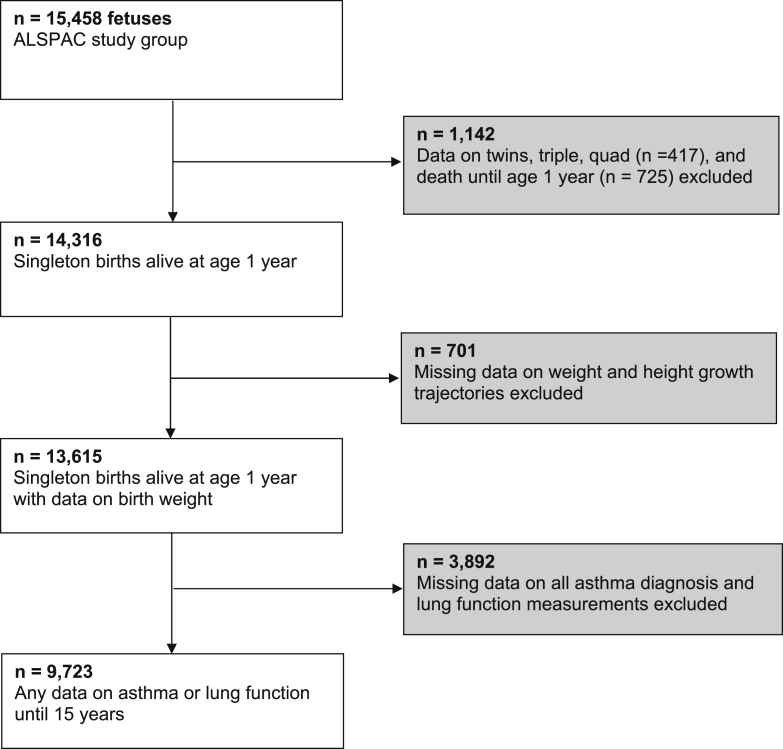
Flow chart of participants in the study.

Subjects were participants in the Avon Longitudinal Study of Parents and Children (ALSPAC) in the United Kingdom, which has been described previously^34^ and on the study's Web site (www.bristol.ac.uk/alspac). In brief, 15,247 pregnant women residing in one of 3 Bristol-based health districts with an expected delivery date of between April 1, 1991, and December 31, 1992, were recruited and gave birth to 14,316 singleton children who were alive at the age of 1 year. Children with no information on either growth trajectories (n = 701) or any asthma outcome (n = 3,892) were excluded, leaving a total of 9,723 children included in the current analyses (see Fig E1 in this article's Online Repository at www.jacionline.org). Ethical approval for the study was obtained from the ALSPAC Law and Ethics Committee and local research ethics committees. Witten informed consent was obtained from all participants and their parents or guardians.

### Growth trajectories

Height and weight measurements were available from birth up to age 10 years from a variety of sources (see the Methods section and Table E1 in this article's Online Repository at www.jacionline.org for full details). Linear spline multilevel models were used to estimate trajectories of height and weight. The models estimate mean and person-specific birth weight or length and mean and person-specific rates of weight or height growth between 0 and 3 months, 3 months and 1 year, 1 and 3 years, 3 and 7 years, and 7 and 10 years of age and are described in full elsewhere.^35^ Early growth was defined as growth between birth and the age of 1 year, midchildhood growth as growth between the ages of 1 and 7 years, and late childhood growth as growth between 7 and 10 years of age. We generated SD scores (*z* scores) for birth weight and length and rate of weight/height growth in each period of childhood by subtracting the mean from the person-specific value and dividing by the SD. These SD scores for birth weight/length and rates of growth are used as exposures in our analyses.

### Asthma and lung function

Current asthma status was obtained at the ages of 8, 14, and 17 years. Current asthma was defined as a reported doctor's diagnosis of asthma ever and reported wheezing, asthma, or use of asthma medication in the previous 12 months. Skin prick test reactivity was determined at the age of 7 years. A child was deemed to react to an allergen (grass, house dust, or cat) if their wheal and/or flare responses were 2 mm or greater and they had no reaction to the negative control. Bronchial hyperresponsiveness, unselected for asthma or wheezing, was measured at the ages of 8 and 15 years.^36^ At age 8 years, we tested the provoking dose of methacholine causing a decrease in FEV_1_ from baseline. The dose-response slope was calculated by fitting a linear function to the plot of percentage decrease from baseline. We dichotomized bronchial responsiveness using the highest tertile as responders and the rest as nonresponders. At age 15 years, we defined bronchial reversibility as a change of equal to or greater than 12% between FEV_1_ before and after inhalation of a standard dose (400 μg) of salbutamol.^37^ Spirometry (Vitalograph 2120; Vitalograph, Maids Moreton, United Kingdom) was performed at 8 and 15 years of age according to American Thoracic Society standards.^38^ Lung function measurements (FEV_1_, forced vital capacity [FVC], forced expiratory flow between 25% and 75% [FEF_25-75_], FEV_1_/FVC ratio, and FEF_25-75_/FVC ratio) were converted into sex-, age-, and height-adjusted *z* scores (see Table E1 for time points of outcomes).^39^

### Covariates

Maternal age, highest qualification, body mass index, parity, and a history of asthma or atopy were reported in questionnaires at 12 weeks of gestation, and smoking during pregnancy was assessed at 18 weeks of gestation by using self-completion questionnaires sent to the mothers. Maternal anxiety during pregnancy was measured at 32 weeks of pregnancy and was defined as the highest quartile of the Crown-Crisp Experiential Index.^40^ Children's gestational age and sex were obtained from birth records. Breast-feeding status at age 8 months was obtained from maternal self-completion questionnaires.

### Statistical analysis

We used logistic regression models to assess associations between growth trajectories and current asthma, atopy, and bronchial responsiveness or reversibility. Linear regression models were used to assess associations of growth trajectories with lung function measurements. Analyses were adjusted for potential confounders, including maternal age, body mass index, anxiety, education, history of asthma or atopy, smoking habits, parity, and the child's sex, gestational age at birth, and breast-feeding status. Models of weight gain were additionally adjusted for birth weight, and preceding rates of height-adjusted weight growth trajectories and models of height gain were additionally adjusted for preceding rates of height growth trajectories and birth weight. Models for current asthma or lung function were additionally adjusted for previous current asthma or lung function measurements. In addition, body mass index at the age of outcome assessment was added as an interaction to explore potential effect modification on the associations of childhood growth with asthma and lung function.

Missing data in confounders were imputed by using multiple imputations. Percentages of missing values within the population for analysis were lower than or near 10%, except for maternal body mass index (13.1%), anxiety (13.6%), and child's breast-feeding duration (11.5%). Ten new data sets were created by means of imputation based on all covariates, determinants, and outcomes in the model.^41^ All data sets were analyzed separately, after which results were combined. No differences in results were observed between analyses with imputed missing data or complete cases only. Therefore we present only results based on imputed data sets. Statistical analyses were performed with the Statistical Package of Social Sciences version 19.0 for Windows (SPSS, Chicago, Ill).

## Results

Characteristics of mothers and their children are presented in Table I. Children were born at a median gestational age of 40 weeks (95% range, 35-42 weeks), with an average birth weight of 3436 grams (SD, 524 grams). Current asthma was reported in 13.9%, 13.2%, and 15.3% of the children at the age of 8, 14, and 17 years. All covariates differed between those included and those excluded from this study, apart from maternal history of asthma (see Table E2 in this article's Online Repository at www.jacionline.org).

### Childhood growth with asthma

We observed no evidence of an association between higher birth length or weight and current asthma (Table II). Height growth in midchildhood tended to be negatively associated with current asthma at age 8 years, with the strongest evidence of association for height gain between 3 and 7 years of age and asthma at 8 years of age (odds ratio [OR], 0.75 [95% CI, 0.66-0.86] per SD increase). More rapid weight gain during early childhood tended to be positively associated with current asthma, with the most consistent associations observed for weight gain between 0 and 3 months of age and asthma at 8 and 17 years of age (OR, 1.10 [95% CI, 1.02-1.19] and 1.18 [95% CI, 1.01-1.37], respectively; Table II). We did not find strong evidence that current body mass index modified the association of childhood growth with asthma. *P* values for the cross-product of growth measurements with body mass index were greater than .05. No associations were observed between any growth measures and skin prick test reactivity (see Table E3 in this article's Online Repository at www.jacionline.org).

### Childhood growth with bronchial responsiveness

We observed no evidence of an association between higher birth length or weight and bronchial hyperresponsiveness (Table III). Also, no evidence was found for associations between height gain in early childhood, midchildhood, or late childhood and bronchial responsiveness or reversibility at 8 and 15 years of age, respectively. Higher weight gain in early childhood (between 0 and 3 and 3 and 12 months of age only) was associated with an increased risk of bronchial responsiveness to methacholine at 8 years (OR, 1.11 [95% CI, 1.03-1.20] and 1.09 [95% CI, 1.00-1.19], respectively, per SD increase) and bronchial responsiveness to salbutamol at 15 years (OR, 1.14 [95% CI, 1.00-1.31] and 1.24 [95% CI, 1.07-1.42], respectively, per SD increase). No strong evidence was observed for effect modification of childhood growth with current body mass index on bronchial responsiveness or reversibility (*P* for interaction > .05).

### Childhood growth with lung function

**Fig 1:**
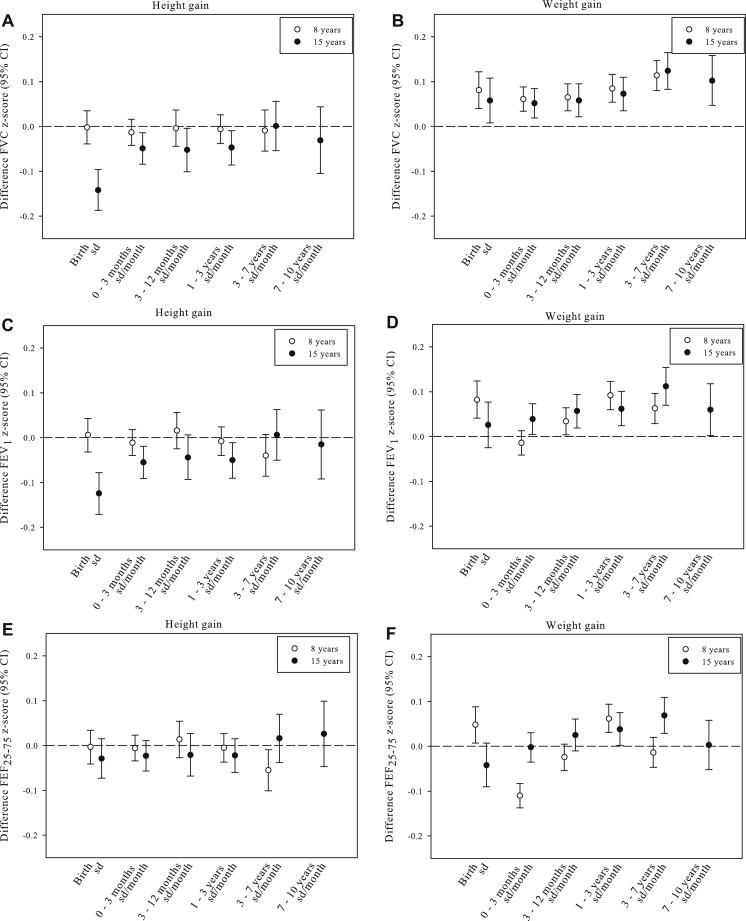

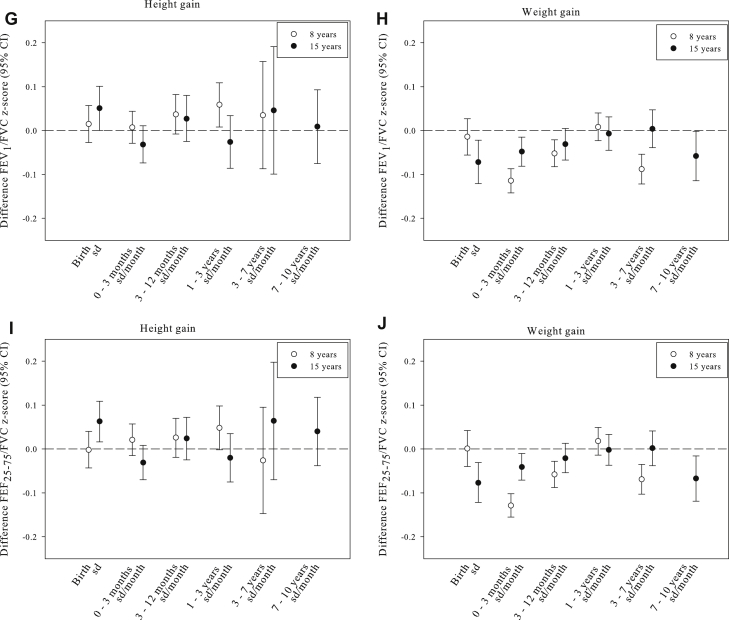
Growth (height and weight) with lung function measures of FVC (**A** and **B**), FEV_1_ (**C** and **D**), FEF_25-75_ (**E** and **F**), and FEV_1_/FVC (**G** and **H**) and FEF_25-75_/FVC (**I** and **J**) ratios. Values are differences in *z* score lung function (95% CIs). *Z* scores were calculated for sex, age, and height at the time of measurement. FEV_1_/FVC and FEF_25-75_/FVC sex-adjusted *z* scores were additionally adjusted for age and height of measurement. Models are adjusted for maternal age, education level, body mass index, parity, smoking during pregnancy, anxiety, history of asthma, and the child's sex, gestational age, and breast-feeding duration. Models of weight were additionally adjusted for preceding height and weight growth trajectories, and models of height were additionally adjusted for preceding height growth trajectories and birth weight. Models for lung function at 15 years of age were additionally adjusted for lung function measures at age 8 years.

Fig 1 and Table E4 in this article's Online Repository at www.jacionline.org show the associations of height and weight trajectories with lung function measurements at 8 and 15 years of age. Higher birth length was associated with a lower FVC and FEV_1_ *z* score at age 15 years (−0.14 [95% CI, −0.19 to −0.09] and −0.12 [95% CI, −0.17 to −0.08] per SD increase, respectively; Fig 1, *A* and *C*). Higher birth length was also associated with higher FEV_1_/FVC and FEF_25-75_/FVC ratios (0.05 [95% CI, 0.00-0.10] and 0.06 [95% CI, 0.02-0.11] per SD increase, respectively; Fig 1, *G* and *I*). After birth, more rapid height gain in early childhood and midchildhood was most consistently associated with a lower FVC and FEV_1_ values at age 15 years but not with other lung function variables or ratios or with lung function at age 8 years (Fig 1, *A-I*).

Higher birth weight was most strongly associated with higher FVC, FEV_1_, and FEF_25-75_ *z* scores at age 8 years (0.08 [95% CI, 0.04-0.12], 0.08 [95% CI, 0.04-0.12], and 0.05 [95% CI, 0.01-0.09] per SD increase, respectively) and with higher FVC values at age 15 years only (0.06 [95% CI, 0.01-0.11]; Fig 1, *B*, *D*, and *F*). Also, higher birth weight was associated with reduced FEV_1_/FVC and FEF_25-75_/FVC ratios at age 15 years (Fig 1, *H* and *J*). After birth, more rapid weight growth throughout childhood was associated with higher FVC and FEV_1_ values, with the greatest effect estimates for weight gain in midchildhood and FVC and FEV_1_ values at age 15 years (0.12 [95% CI, 0.08-0.17] and 0.11 [95% CI, 0.07-0.15], *z* score per SD, respectively; Fig 1, *B* and *D*). For the other lung function variables, more rapid weight gain in early childhood was associated with a decreased FEF_25-75_ value at 8 years of age only (Fig 1, *F*). We observed lower FEV_1_/FVC and FEF_25-75_/FVC ratios at the ages of 8 and 15 years for early rapid weight gain, followed by normal ratios for midchildhood weight gain but lower ratios for late rapid weight gain (Fig 1, *H* and *J*). We observed effect modification of childhood weight growth by current body mass index on lung function (*P* for interaction < .05) but not of childhood height growth (*P* for interaction > .05). Stratified analyses for body mass index showed that the effect estimates of childhood weight growth for FVC and FEV_1_ were larger in the group of children with a normal body mass index compared with the overweight children (see Table E5 in this article's Online Repository at www.jacionline.org).

## Discussion

Our results suggest positive associations of rapid weight growth during early childhood and midchildhood with current asthma, higher weight growth during early childhood with increased bronchial responsiveness or reversibility, and higher weight growth in childhood with higher overall lung volumes but increased measures of obstruction (FEV_1_/FVC and FEF_25-75_/FVC ratios) in childhood. Higher length at birth and height growth in childhood were associated with lower lung volumes but less consistently associated with the other respiratory outcomes.

### Comparison with previous studies

Previous studies of the association of childhood growth with asthma have reported an increased risk of asthma symptoms in preschool children with accelerated growth in early infancy.^10,23^ A previous study that measured asthma at an older age (6 years) showed no evidence for increased risks caused by changes in growth using similarly defined growth trajectories as in our study. However, those authors did report increased risks of ever wheezing in those with higher weight growth in early childhood.^24^ Differences in results with our study might be explained by differences in the study populations (general population, term-born children only) and the age at which asthma was measured (early childhood, midchildhood, or late childhood). A meta-analysis on body mass index gain in early childhood and midchildhood suggested that more rapid body mass index gain in early childhood but not thereafter was associated with an increased incidence of asthma at age 6 years,^22^ which is consistent with our findings about asthma at age 8 years.

To the best of our knowledge, no previous studies have examined the relationship between childhood growth and bronchial responsiveness or reversibility. However, because asthma is associated with bronchial hyperresponsiveness,^42^ the association between early childhood weight gain and the objective measure of bronchial responsiveness is in line with previous studies on growth and asthma outcomes,^9,10,22-24,27^ and this strengthens our conclusions about the association with asthma by using both objective and self-reported outcome measures. Previous studies that measured lung function during early childhood reported lower FEV_0.4_ values in the first months of life in term-born children with greater postnatal weight gain.^21^ Turner et al^27^ showed a negative association of growth between 1 and 12 months of age and lung function change (V′max FRC [maximal flow at functional residual capacity]) during the same period. Only a tendency toward an association of growth with lower FEF_25-75_ values at 11 years of age was observed.^27^ Our findings were in line with these results. In contrast, Canoy et al^19^ showed in adults that weight gain during the first year was positively associated with adult lung function independent of birth weight. Additionally, we showed in a large number of subjects that weight gain in midchildhood and late childhood was associated with lung function independent of birth weight and weight gain in early childhood.

### Interpretation of results

The most prominent and novel findings in this study are the positive associations of weight gain in early childhood, specifically weight gain in the first 3 months of life, and lung function changes at 8 and 15 years of age. This early postnatal period has been observed previously to be important for the development of asthma symptoms and decreased lung function up to preschool age.^10,21,23,24^ Our results suggest that the effects of rapid weight gain in the first 3 months of life on asthma and bronchial hyperresponsiveness persist until adolescence. Weight gain between 0 and 3 months of age was associated with asthma at both 8 and 17 years of age, whereas weight gain between 3 and 12 months of age was only associated with asthma at age 8 years. This might be due to a different underlying mechanism between these intervals, such as early developmental influences resulting in persistent changes in airway or immune development after more rapid growth in the first months after birth. Whereas, more rapid growth and multiple other exposures of influence after these first months leads to modifiable changes. However, this also might be a chance finding or caused by the smaller number of children in the older age group. Therefore these associations need to be replicated in other studies. Additionally, rapid weight growth in midchildhood and late childhood was associated with changes in lung function variables. The underlying mechanisms of rapid weight gain in childhood on asthma and lung function outcomes are unclear and should be assessed in future studies. We speculate that abnormal growth and development of the lungs, possibly with mismatch between airway and alveolar growth or immunologic and inflammatory effects with lung and airway remodeling, might play a role.^29,32,43^ Both airways and alveoli continue to develop, at least until adolescence.^44^ Therefore exposures, including growth, during childhood are likely to influence this development. Our results suggest that growth in the first months after birth is the most important in the association with asthma and lung function because most rapid developmental and growth changes occur during this time. Growth after this early period also might influence lung function but might not completely reverse the early effects. Also, higher leptin levels have been associated with lower lung function in childhood, suggesting a possible mechanism mediated by adiposity-related hormones that might influence pulmonary growth factors, such as vascular endothelial growth factor, transforming growth factor, and insulin-like growth factor.^45^ Also, unmeasured factors determining early growth might influence respiratory health in adolescence. The FEV_1_/FVC ratio is a measure of obstruction, and decreased values are a feature of asthma. We observed increases in FVC and FEV_1_ values in association with rapid early weight gain but a lower FEV_1_/FVC ratio, which would be consistent with greater influence of early rapid weight gain on lung volume than airway growth. Because weight gain in infancy is proportionally greater than in subsequent years, effects of rapid weight gain on an imbalance between FEV and FVC values might be most influenced during this specific period. The FEF_25-75_/FVC ratio has also been suggested as a measure of dysanapsis in which airways are small in relation to total lung capacity,^46^ and therefore our finding of rapid weight gain associations with lower FEF_25-75_/FVC ratios would be consistent with this explanation. The associations of early childhood and midchildhood height growth and decreased FVC and FEV_1_ values at the age of 15 years could also point to dysanapsis; however, we did not observe an association of height growth with the FEV_1_/FVC ratio. Therefore height growth is less likely to be associated with a mismatch between airway and alveolar growth.

Although we did not observe an association of growth with skin prick test reactivity, another possible explanation for effects of rapid weight gain on lung function is through influence of adipose tissue on the developing immune system through secretion of immunologically active factors, including adipokines and chemokines, which stimulate release of TNF-α and interleukins.^47^ In mice leptin has been shown to enhance airway responsiveness, suggesting an immunomodulatory role,^48^ and the effect has also been reported in human subjects, although results are inconsistent.^49-51^ If this potential underlying mechanism is a factor in the association between growth and asthma, we suggest that not only obesity but also weight gain in normal and overweight children leads to increased leptin levels. We observed no evidence that body mass index modified associations of childhood growth with asthma and bronchial responsiveness or reversibility or on the association of childhood height growth with lung function. We observed effect modification of childhood weight growth by current body mass index on lung function (*P* for interaction < .05). Stratified analyses for body mass index showed that the effect estimates of childhood weight growth for FVC and FEV_1_ values were larger in the group of children with a normal body mass index compared with overweight children (see Table E2). This suggests that the development of lung and airway volumes of overweight subjects are less influenced by weight gain in childhood than lung and airway volumes of normal-weight children. The underlying mechanisms should be studied in future research. Finally, a common unknown factor that increases weight gain and is also responsible for a higher risk of respiratory morbidity, such as shared genetic risk, might be involved.^52^

### Strengths and limitations

This study was embedded in a population-based prospective cohort study, with a large number of subjects being studied from pregnancy onward and detailed and prospectively acquired information about growth and respiratory morbidity. Modeled growth trajectories for this population enabled us to take account of different timings and numbers of measurements between children. Additionally, previous changes in weight and height were considered in the models, and therefore changes in the time intervals reflect growth during that specific interval independent of earlier growth and not simply catch-up or down growth after early aberrant growth. Because puberty can influence pulmonary physiology, an important limitation of our growth models is that they do not continue during puberty. Growth and puberty have a bidirectional relationship, which makes disentangling their effects complex. Further research is needed to explore the effects of growth during and after puberty with respiratory health. Lung function measurements were made by using the same methods at 2 time points, and methacholine challenge or bronchodilator reversibility were used to evaluate bronchial responsiveness, producing objective respiratory outcomes. After Bonferroni corrections for multiple testing (*P* < .00625), only the associations of growth with asthma did not hold. However, statistical corrections for multiple comparisons seem conservative because all exposures and outcomes are correlated. We adjusted for a large number of confounders.

Loss to follow-up data in the ALSPAC cohort is associated with social deprivation but not with atopic predisposition.^53^ Nonresponse and loss to follow-up would lead to biased effect estimates if associations of childhood growth with respiratory outcomes differed between those included and not included in the analyses. This is unlikely but difficult to study. Therefore we believe that the differences in covariates between those included and excluded did not influence the effect estimates but might have affected the generalizability of the observed effects.

Also, we were unable to take fetal growth into account. Growth in childhood might be the result of various fetal growth patterns that could underlie associations of growth in childhood with asthma and lung function. However, previous studies showed inconsistent effects of fetal growth with respiratory outcomes.^8,10^

In conclusion, our results suggest that rapid weight growth during specific intervals in childhood is associated with current asthma, increased bronchial responsiveness and reversibility, and higher lung volumes and measures of obstruction. Rapid length growth was only associated with lower overall lung volume. Therefore changes in weight, especially early weight growth, appear to be important in lung development. Further studies are needed to replicate these findings and to explore the underlying mechanisms of the effect of growth in specific periods on respiratory health and differential lung growth.

**Key message**

• Faster weight growth in early childhood is positively associated with asthma and bronchial hyperresponsiveness during adolescence.

## Figures and Tables

**Table E1:** Time points at which information on determinants and outcomes were obtained

Age (y)	Determinant	Outcomes
1	Growth model	
2	Growth model	
3	Growth model	
4	Growth model	
5	Growth model	
6	Growth model	
7	Growth model	
8	Growth model	Asthma
		Bronchial responsiveness (methacholine)
		Spirometry
9	Growth model	
10	Growth model	
11		
12		
13		
14		Asthma
15		Bronchial reversibility (salbutamol)
		Spirometry
16		
17		Asthma

**Table E2:** Characteristics of included and excluded children and their mothers

Maternal characteristics	Included (n = 9723)	Excluded (n = 4593)	*P* value difference
Age (y)			
<20	4.3 (404)	14.2 (592)	<.001
20-24	19.8 (1873)	32.6 (1260)	
	41.8 (3945)	33.2 (1387)	
30-34	26.1 (2466)	15.5 (646)	
≥35	8.0 (756)	4.5 (188)	
Missing	2.9 (279)	9.1 (420)	
Body mass index (kg/m^2^)			
<20	18.2 (1541)	22.1 (614)	<.001
20-24	61.3 (5183)	56.8 (1581)	
25-29	15.2 (1282)	15.0 (418)	
≥30	5.3 (448)	6.1 (171)	
Missing	13.1 (1269)	39.4 (1809)	
Education (%)			
Low/medium	60.3 (5454)	77.5 (2366)	<.001
Higher	39.7 (3,596)	22.5 (686)	
Missing	6.9 (673)	33.6 (1541)	
History of asthma (%)			
No	88.8 (8042)	88.2 (2718)	.363
Yes	11.2 (1018)	11.8 (365)	
Missing	6.8 (663)	32.9 (1510)	
Anxiety during pregnancy (%)			
No	73.3 (6162)	66.4 (2033)	<.001
Yes	26.7 (2241)	33.6 (1030)	
Missing	13.6 (1220)	33.3 (1530)	
Smoking during pregnancy (%)			
No	84.0 (7753)	70.4 (2535)	<.001
Yes	16.0 (1472)	29.6 (1065)	
Missing	5.1 (498)	21.6 (993)	
Parity (%)			
0	46.0 (4181)	42.1 (1479)	<.001
≥1	54.0 (4909)	57.9 (2031)	
Missing	6.5 (633)	29.6 (1083)	
Child characteristics			
Female sex (%)	49.5 (4814)	47.1 (2165)	.008
Gestational age at birth (wk)	40.0 (35.0-42.0)	40.0 (34.0-42.0)	.006
Birth weight (g)	3438 (532)	3362 (556)	<.001
Breast-feeding duration (%)			
Never	23.3 (1999)	38.6 (759)	<.001
<3 mo	22.9 (1972)	22.6 (465)	
3-6 mo	17.1 (1472)	14.8 (305)	
≥6 mo	36.8 (3163)	24.0 (494)	
Missing	11.5 (1117)	55.2 (2534)	

Values are means (SDs), medians (2.5th-97.5th percentiles), or percentages (absolute numbers). Differences were tested by using the Student *t* test for continuous variables, the χ^2^ test for categorical variables, and the Mann-Whitney test for the not normally distributed variable (gestational age at birth).

**Table E3:** Growth (height and weight) with skin prick tests

	OR (95% CI [n = 6439])	*P* value
Height		
Birth length (SD)	0.93 (0.84-1.02)	.13
0-3 mo (SD/mo)	0.99 (0.92-1.06)	.73
3-12 mo (SD/mo)	1.02 (0.92-1.13)	.72
1-3 y (SD/mo)	0.96 (0.89-1.04)	.35
3-7 y (SD/mo)	0.97 (0.86-1.10)	.66
7-10 y (SD/mo)	—	
Weight		
Birth weight (SD)	1.07 (0.96-1.19)	.21
0-3 mo (SD/mo)	1.03 (0.96-1.11)	.37
3-12 mo (SD/mo)	0.98 (0.91-1.06)	.68
1-3 y (SD/mo)	1.08 (0.99-1.17)	.08
3-7 y (SD/mo)	1.02 (0.93-1.11)	.69
7-10 y (SD/mo)	—	

Values are ORs (95% CIs) for positive skin prick test responses. Models are adjusted for maternal age, education level, history of asthma, body mass index, parity, smoking during pregnancy, anxiety, and the child's sex, gestational age, breast-feeding duration, and previous height or weight gain. Models of weight were additionally adjusted for preceding height and weight growth trajectories, and models of height were additionally adjusted for preceding height growth trajectories and birth weight.

**Table E4:** Growth (height and weight) with lung function measures

	Mean difference (95% CI)			
	Height growth		Weight growth	
	8 y of age	15 y of age	8 y of age	15 y of age
FVC				
Birth	−0.002 (−0.039 to 0.035)	−0.142 (−0.187 to −0.096)	0.081 (0.040 to 0.122)	0.058 (0.008 to 0.108)
0-3 mo	−0.013 (−0.042 to 0.016)	−0.049 (−0.084 to −0.014)	0.061 (0.034 to 0.088)	0.052 (0.019 to 0.085)
3-12 mo	−0.004 (−0.044 to 0.037)	−0.052 (−0.101 to −0.004)	0.065 (0.035 to 0.095)	0.058 (0.022 to 0.095)
1-3 y	−0.006 (−0.038 to 0.026)	−0.047 (−0.086 to −0.009)	0.085 (0.054 to 0.116)	0.073 (0.035 to 0.110)
3-7 y	−0.009 (−0.055 to 0.037)	0.001 (−0.054 to 0.056)	0.114 (0.080 to 0.147)	0.124 (0.083 to 0.165)
7-10 y		−0.031 (−0.105 to 0.044)		0.102 (0.047 to 0.158)
FEV_1_				
Birth	0.006 (−0.032 to 0.043)	−0.124 (−0.171 to −0.078)	0.082 (0.041 to 0.124)	0.026 (−0.025 to 0.077)
0-3 mo	−0.011 (−0.04 to 0.018)	−0.055 (−0.091 to −0.019)	−0.014 (−0.041 to 0.013)	0.039 (0.005 to 0.073)
3-12 mo	0.016 (−0.025 to 0.056)	−0.044 (−0.093 to 0.006)	0.034 (0.004 to 0.064)	0.057 (0.019 to 0.094)
1-3 y	−0.008 (−0.040 to 0.024)	−0.05 (−0.090 to −0.011)	0.092 (0.06 to 0.123)	0.062 (0.024 to 0.101)
3-7 y	−0.04 (−0.086 to 0.007)	0.006 (−0.050 to 0.063)	0.063 (0.029 to 0.096)	0.112 (0.070 to 0.154)
7-10 y		−0.015 (−0.092 to 0.062)		0.060 (0.002 to 0.118)
FEF_25-75_				
Birth	−0.003 (−0.041 to 0.034)	−0.029 (−0.073 to 0.015)	−0.014 (−0.056 to 0.027)	−0.072 (−0.121 to −0.022)
0-3 mo	−0.006 (−0.034 to 0.023)	−0.023 (−0.057 to 0.011)	−0.114 (−0.142 to −0.087)	−0.048 (−0.081 to −0.015)
3-12 mo	0.014 (−0.027 to 0.054)	−0.021 (−0.068 to 0.027)	−0.052 (−0.082 to −0.021)	−0.031 (−0.067 to 0.005)
1-3 y	−0.005 (−0.037 to 0.027)	−0.022 (−0.060 to 0.015)	0.008 (−0.023 to 0.040)	−0.007 (−0.045 to 0.031)
3-7 y	−0.055 (−0.101 to −0.009)	0.016 (−0.038 to 0.07)	−0.088 (−0.122 to −0.054)	0.004 (−0.039 to 0.047)
7-10 y		0.026 (−0.047 to 0.099)		−0.058 (−0.114 to −0.002)
FEV_1_/FVC ratio				
Birth	0.015 (−0.027 to 0.057)	0.051 (0.000328 to 0.101)	0.001 (−0.040 to 0.042)	−0.077 (−0.122 to −0.031)
0-3 mo	0.007 (−0.029 to 0.044)	−0.032 (−0.074 to 0.011)	−0.129 (−0.155 to −0.102)	−0.041 (−0.071 to −0.010)
3-12 mo	0.037 (−0.008 to 0.082)	0.027 (−0.025 to 0.080)	−0.058 (−0.088 to −0.028)	−0.021 (−0.054 to 0.013)
1-3 y	0.059 (0.008 to 0.109)	−0.026 (−0.086 to 0.034)	0.018 (−0.014 to 0.049)	−0.002 (−0.037 to 0.033)
3-7 y	0.035 (−0.087 to 0.157)	0.046 (−0.099 to 0.191)	−0.069 (−0.103 to −0.035)	0.002 (−0.038 to 0.041)
7-10 y		0.009 (−0.075 to 0.093)		−0.067 (−0.119 to −0.016)
FEF_25-75_/FVC ratio				
Birth	−0.002 (−0.043 to 0.04)	0.063 (0.016 to 0.109)	0.048 (0.007 to 0.088)	−0.042 (−0.09 to 0.007)
0-3 mo	0.021 (−0.015 to 0.057)	−0.031 (−0.07 to 0.008)	−0.11 (−0.137 to −0.083)	−0.002 (−0.035 to 0.03)
3-12 mo	0.026 (−0.019 to 0.070)	0.024 (−0.025 to 0.072)	−0.024 (−0.054 to 0.005)	0.025 (−0.01 to 0.061)
1-3 y	0.048 (−0.002 to 0.098)	−0.020 (−0.075 to 0.035)	0.062 (0.031 to 0.094)	0.038 (0.002 to 0.075)
3-7 y	−0.026 (−0.147 to 0.095)	0.064 (−0.07 to 0.198)	−0.014 (−0.047 to 0.02)	0.069 (0.029 to 0.109)
7-10 y		0.04 (−0.038 to 0.118)		0.003 (−0.052 to 0.058)

Values are differences in *z* score lung function (95% CIs). *Z* scores were calculated for sex, age, and height at the time of measurement. Models are adjusted for maternal age, education level, body mass index, parity, smoking during pregnancy, anxiety, history of asthma, and the child's sex, gestational age, and breast-feeding duration. Models of weight were additionally adjusted for preceding height and weight growth trajectories, and models of height were additionally adjusted for preceding height growth trajectories and birth weight. Models for lung function at 15 years of age were additionally adjusted for lung function measures at age 8 years.

**Table E5:** Weight growth trajectories and lung function in strata of current body mass index

	FVC (*z* score)	*P* value	*P* value for interaction	FEV_1_ (*z* score)	*P* value	*P* value for interaction	FEF_25-75_ (*z* score)	*P* value	*P* value for interaction
8 y of age									
Normal BMI at 8 y									
Birth weight (SD)	0.072 (0.020 to 0.124)	.006	.85	0.062 (0.010 to 0.115)	.02	.77	−0.033 (−0.086 to 0.019)	.21	.99
0-3 mo (SD/mo)	0.056 (0.021 to 0.091)	.001	.13	−0.007 (−0.041 to 0.028)	.71	.03	−0.103 (−0.137 to −0.068)	<.001	.25
3-12 mo (SD/mo)	0.062 (0.023 to 0.101)	.002	.42	0.048 (0.008 to 0.087)	.02	.02	−0.024 (−0.063 to 0.015)	.23	.01
1-3 y (SD/mo)	0.086 (0.043 to 0.128)	<.001	.38	0.089 (0.046 to 0.132)	<.001	.52	0.007 (−0.036 to 0.050)	.76	.49
3-7 y (SD/mo)	0.293 (0.217 to 0.368)	<.001	<.001	0.266 (0.190 to 0.343)	<.001	<.001	−0.084 (−0.161 to −0.006)	.03	.01
7-10 y (SD/mo)									
Overweight at 8 y									
Birth weight (SD)	0.087 (0.014 to 0.160)	.02	.85	0.101 (0.027 to 0.175)	.01	.77	0.024 (−0.052 to 0.100)	.53	.99
0-3 mo (SD/mo)	0.035 (−0.014 to 0.083)	.16	.13	−0.025 (−0.074 to 0.025)	.33	.03	−0.086 (−0.137 to −0.035)	.001	.25
3-12 mo (SD/mo)	0.039 (−0.013 to 0.092)	.14	.42	−0.002 (−0.055 to 0.051)	.95	.02	−0.080 (−0.134 to −0.026)	.004	.01
1-3 y (SD/mo)	0.061 (0.008 to 0.114)	.03	.38	0.096 (0.043 to 0.150)	.001	.52	0.051 (−0.004 to 0.107)	.07	.49
3-7 y (SD/mo)	0.049 (−0.009 to 0.106)	.10	<.001	−0.010 (−0.069 to 0.049)	.733	<.001	−0.091 (−0.151 to −0.030)	.003	.01
7-10 y (SD/mo)									
15 y of age									
Normal BMI at 15 y									
Birth weight (SD)	0.076 (0.021 to 0.131)	.007	.09	0.043 (−0.015 to 0.101)	.15	.22	−0.080 (−0.185 to 0.026)	.14	.64
0-3 mo (SD/mo)	0.022 (−0.015 to 0.059)	.25	.06	0.017 (−0.022 to 0.056)	.39	.11	−0.019 (−0.056 to 0.019)	.32	.08
3-12 mo (SD/mo)	0.037 (−0.005 to 0.078)	.08	.82	0.033 (−0.010 to 0.077)	.13	.85	0.016 (−0.025 to 0.057)	.45	.46
1-3 y (SD/mo)	0.055 (0.011 to 0.099)	.01	.01	0.046 (0.000 to 0.093)	.05	.22	0.035 (−0.009 to 0.079)	.12	.17
3-7 y (SD/mo)	0.192 (0.131 to 0.252)	<.001	<.001	0.218 (0.154 to 0.281)	<.001	<.001	0.178 (0.117 to 0.238)	<.001	<.001
7-10 y (SD/mo)	0.110 (0.041 to 0.180)	.002	<.001	0.072 (−0.002 to 0.145)	.06	<.001	−0.008 (−0.078 to 0.061)	.81	<.001
Overweight at 15 y									
Birth weight (SD)	−0.036 (−0.146 to 0.074)	.52	.09	−0.052 (−0.161 to 0.057)	.35	.22	−0.032 (−0.087 to 0.023)	.26	.64
0-3 mo (SD/mo)	0.127 (0.055 to 0.200)	.001	.06	0.098 (0.025 to 0.170)	.008	.11	0.050 (−0.021 to 0.121)	.17	.08
3-12 mo (SD/mo)	0.055 (−0.023 to 0.134)	.17	.82	0.079 (0.001 to 0.156)	.048	.85	0.036 (−0.040 to 0.112)	.35	.46
1-3 y (SD/mo)	0.047 (−0.029 to 0.124)	.23	.01	0.062 (−0.014 to 0.138)	.11	.22	0.034 (−0.041 to 0.108)	.37	.17
3-7 y (SD/mo)	−0.057 (−0.137 to 0.022)	.16	<.001	−0.041 (−0.120 to 0.038)	.31	<.001	−0.051 (−0.128 to 0.026)	.19	<.001
7-10 y (SD/mo)	0.036 (−0.070 to 0.143)	.51	<.001	0.040 (−0.064 to 0.145)	.45	<.001	0.043 (−0.060 to 0.146)	.41	<.001

Values are differences in *z* score lung function (95% CIs). *Z* scores were calculated for sex, age, and height at the time of measurement. Models are adjusted for maternal age, education level, body mass index, parity, smoking during pregnancy, anxiety, history of asthma, and the child's sex, gestational age, and breast-feeding duration. Models of weight were additionally adjusted for preceding height and weight growth trajectories, and models of height were additionally adjusted for preceding height growth trajectories and birth weight. Models for lung function at age 15 years were additionally adjusted for lung function measures at age 8 years.

**Table I:** Characteristics of mothers and their children (n = 9723)

Maternal characteristics	Observed	Imputed
Age (y)		
<20	4.3 (404)	4.3 (417)
20-24	19.8 (1873)	19.8 (1928)
25-29	41.8 (3945)	41.8 (4066)
30-34	26.1 (2466)	26.1 (2537)
≥35	8.0 (756)	8.0 (776)
Missing	2.9 (279)	—
Body mass index (kg/m^2^)		
<20	18.2 (1541)	18.7 (1815)
20-24	61.3 (5183)	59.5 (5793)
25-29	15.2 (1282)	16.7 (1623)
≥30	5.3 (448)	5.1 (492)
Missing	13.1 (1,269)	—
Education (%)		
Low/medium	60.3 (5454)	60.7 (5902)
Higher	39.7 (3596)	39.3 (3821)
Missing	6.9 (673)	—
History of asthma (%)		
No	88.8 (8042)	88.7 (8629)
Yes	11.2 (1018)	11.3 (1094)
Missing	6.8 (663)	—
Anxiety during pregnancy (%)		
No	73.3 (6162)	73.3 (7126)
Yes	26.7 (2241)	26.7 (2597)
Missing	13.6 (1220)	—
Smoking during pregnancy (%)		
No	84.0 (7753)	83.9 (8159)
Yes	16.0 (1472)	16.1 (1564)
Missing	5.1 (498)	—
Parity (%)		
0	46.0 (4181)	46.1 (4486)
≥1	54.0 (4909)	53.9 (5237)
Missing	6.5 (633)	—
Child characteristics		
Female sex (%)	49.5 (4814)	49.5 (4814)
Gestational age at birth (wk)	40.0 (35.0-42.0)	40.0 (35.0-42.0)
Birth weight (g)	3438 (532)	3436 (524)
Breast-feeding duration (%)		
Never	23.3 (1999)	23.7 (2308)
<3 mo	22.9 (1972)	23.0 (2236)
3-6 mo	17.1 (1472)	17.1 (1664)
≥6 mo	36.8 (3163)	36.2 (3515)
Missing	11.5 (1117)	—

Values are means (SDs), medians (2.5th-97.5th percentiles), or percentages (absolute numbers). Gestational age at birth was missing for 2.9% (n = 279), and birth weight was missing for 3.9% (n = 378).

**Table II:** Growth trajectories and current asthma

	Current asthma					
	8 y		14 y		17 y	
	n = 7794	*P* value	n = 5590	*P* value	n = 3531	*P* value
Height						
Birth length (SD)	0.97 (0.88-1.08)	.60	0.97 (0.84-1.12)	.66	0.94 (0.76-1.16)	.48
0-3 mo (SD/mo)	0.98 (0.91-1.06)	.57	0.97 (0.87-1.09)	.59	1.05 (0.89-1.24)	.55
3-12 mo (SD/mo)	1.02 (0.91-1.14)	.76	0.93 (0.79-1.08)	.32	1.05 (0.85-1.30)	.66
1-3 y (SD/mo)	0.91 (0.84-0.99)	.03	0.96 (0.85-1.08)	.48	0.91 (0.77-1.09)	.31
3-7 y (SD/mo)	0.75 (0.66-0.86)	<.001	1.10 (0.92-1.31)	.32	1.14 (0.88-1.47)	.32
7-10 y (SD/mo)	—		1.06 (0.84-1.35)	.62	0.81 (0.57-1.14)	.23
Weight						
Birth weight (SD)	0.99 (0.89-1.10)	.81	0.97 (0.83-1.13)	.69	1.13 (0.91-1.41)	.27
0-3 mo (SD/mo)	1.09 (1.02-1.17)	.02	0.97 (0.88-1.08)	.61	1.18 (1.01-1.37)	.03
3-12 mo (SD/mo)	1.10 (1.02-1.19)	.02	1.10 (0.98-1.24)	.10	0.89 (0.75-1.06)	.18
1-3 y (SD/mo)	1.11 (1.02-1.20)	.02	1.03 (0.91-1.16)	.68	1.03 (0.87-1.23)	.72
3-7 y (SD/mo)	1.03 (0.94-1.13)	.57	0.94 (0.82-1.08)	.39	1.04 (0.86-1.26)	.69
7-10 y (SD/mo)	—		1.00 (0.82-1.21)	.97	0.92 (0.70-1.21)	.54

Values are ORs (95% CIs). Models are adjusted for maternal age, education level, history of asthma, body mass index, parity, smoking during pregnancy, anxiety, and the child's sex, gestational age, breast-feeding duration, and previous height or weight gain. Models of weight were additionally adjusted for preceding height and weight growth trajectories, and models of height were additionally adjusted for preceding height growth trajectories and birth weight. Also, models were additionally adjusted for previous current asthma.

**Table III:** Growth trajectories and bronchial responsiveness at age 8 years and bronchial reversibility at age 15 years

	Methacholine responsive at 8 y n = 4389	*P* value	Salbutamol responsive at 15 y n = 3750	*P* value
Height				
Birth length (SD)	1.00 (0.90-1.11)	.98	1.01 (0.84-1.21)	.95
0-3 mo (SD/mo)	0.96 (0.89-1.04)	.31	1.12 (0.97-1.29)	.12
3-12 mo (SD/mo)	0.99 (0.89-1.11)	.88	1.06 (0.87-1.28)	.59
1-3 y (SD/mo)	1.01 (0.92-1.10)	.82	1.04 (0.89-1.21)	.62
3-7 y (SD/mo)	0.96 (0.84-1.10)	.55	1.18 (0.95-1.48)	.14
7-10 y (SD/mo)	—		1.04 (0.77-1.40)	.81
Weight				
Birth weight (SD)	0.94 (0.84-1.06)	.29	0.93 (0.76-1.14)	.47
0-3 mo (SD/mo)	1.11 (1.03-1.20)	.006	1.14 (1.00-1.31)	.05
3-12 mo (SD/mo)	1.09 (1.00-1.19)	.05	1.24 (1.07-1.42)	.003
1-3 y (SD/mo)	0.95 (0.87-1.04)	.23	0.87 (0.75-1.01)	.08
3-7 y (SD/mo)	1.00 (0.91-1.10)	.96	1.09 (0.93-1.28)	.31
7-10 y (SD/mo)	—		0.93 (0.74-1.17)	.52

Values are ORs (95% CIs) of bronchial responsiveness or reversibility. Models are adjusted for maternal age, education level, history of asthma, body mass index, parity, smoking during pregnancy, anxiety, and the child's sex, gestational age, breast-feeding duration, and previous height or weight gain. Models of weight were additionally adjusted for preceding height and weight growth trajectories, and models of height were additionally adjusted for preceding height growth trajectories and birth weight. *Methacholine responsive*, Highest tertile versus lower tertiles; *Salbutamol responsive*, greater than 12% change in FEV_1_ vs less than 12% change.

## Methods

### Growth trajectories

Length and height data for the children were available from several sources. Birth length (crown-heel) was measured with a Harpenden Neonatometer (Holtain, Crosswell, Crymych, United Kingdom) by trained research staff who visited newborns soon after birth (median, 1 day; range, 1 to 14 days). From birth to age 5 years, measurements were available from health visitor records, which form part of standard childcare in the United Kingdom, for the majority of the cohort. On average, up to 4 measurements were taken at 2, 10, 21, and 48 months of age, which has been demonstrated previously to have good accuracy.^E1^ For a random 10% of the cohort, direct measurements from a series of research clinics held between the ages of 4 months and 5 years were also available. At these clinics, crown-heel length for children aged 4 to 25 months was measured with a Harpenden Neonatometer (Holtain), and from 25 months onward, standing height was measured with a Leicester height measure (Seca, Hamburg, Germany). Weight was measured by using a Seca scale. From age 7 years upward, all children were invited to annual clinics, at which time standing height was measured (without shoes) to the last complete millimeter by using the Harpenden stadiometer (Holtain), and Tanita Body Fat Analyses (Model TBF 305) were used for weight measurement. Across all ages, parent-reported child heights and weights were also available from questionnaires. These measurements were comparable with routinely collected child health record height and weight data with no systematic bias.^E1^ Therefore all the above-described height and weight measurements were used to generate growth trajectories for height and weight from children with at least 2 observed measurements.

We used fractional polynomials to find the best-fitting average trajectory and used this to derive approximate knot points for a linear spline model.^E2^ A separate model was created for female and male subjects. We simplified the models with the aim of having the same knot points in female and male subjects for both weight and height and having knot points at round ages in months. Model fit with these models was not appreciably lower than in the optimal model. The defined knot points are 3 months and 1, 3, and 7 years, thereby creating the following growth rate trajectories: 0 to 3 months, 3 months to 1 year, 1 to 3 years, 3 to 7 years, and 7 to 10 years. Growth rates are presented as changes in SD, which were generated by subtracting the mean from the person-specific value and dividing by the SD.

Please note that the study Web site contains details of all the data, which are available through a fully searchable data dictionary at the following Web page: www.bris.ac.uk/alspac/researchers/data-access/data-dictionary.
